# Bibliometric Analysis of the Highest Cited Cosmetic Upper Facial Plastic Surgery Articles Over 50 Years

**DOI:** 10.1093/asjof/ojae123

**Published:** 2024-12-16

**Authors:** Nitin Rangu, Evan Pistone, Jeremy Tan, Thai Do

## Abstract

In this bibliometric analysis, the authors analyze the top 100 (T100) most cited articles on cosmetic upper facial plastic surgery. Throughout this study, the objective of the authors is to delineate the trends in cosmetic upper facial surgeries to identify prevailing techniques, emerging trends, and potential areas of future investigation. The articles were indexed from the Web of Science database and were extracted in a double-blinded manner by 2 independent graders. The search phrase used covered a wide range of cosmetic upper facial plastic surgeries, of which a short sample is included: (“cosmetic*” AND “bleph*”) OR (“cosmetic*” AND “upper eyelid blepharoplasty”) OR (“cosmetic*” AND “lower eyelid blepharoplasty”). In their statistical analysis of the number of citations received in each article in the T100, the authors reveal an average of 55.1 citations (a standard deviation of 38.7). Surgical methods were the most commonly cited unique study area, with 30% of the T100, followed by botulinum toxin and complication management with 29% and 15% of the T100, respectively. The unique study area with the highest average citations received was botulinum toxin, with an average of 64.7 citations. Invasive procedures made up 55% of the T100 articles. The authors found that the late 1990s and 2000s were a burgeoning period of growth in this field and highlight the evolution of many contemporary popular cosmetic procedures over time. Particularly, a growth in minimally invasive procedures was noted, with noted impacts in aesthetics training and research focus.

**Level of Evidence: 4 (Therapeutic):**

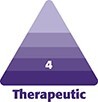

Analyzing citation patterns associated with articles on cosmetic upper facial procedures, the authors provide valuable insights into how this field has evolved over the past 5 decades. This understanding enables researchers and clinicians to better grasp shifts in demand and interest, informing future research focuses. The face plays a pivotal role in shaping personal identity and societal perceptions, motivating individuals to seek cosmetic facial plastic surgery to achieve their desired appearance.^1^ For this discussion, the upper face encompasses the eye and orbit, forehead, and temporal region. Common surgical procedures in this area include blepharoplasty, facial fat grafting, and minimally invasive options such as soft tissue fillers and Botox injections.^2^ Between 2019 and 2022, the popularity of these surgeries increased by 18%, reflecting a growing societal interest in facial modification. Notably, there was a 73% increase in the usage of botulinum toxin for facial procedures during this period, although specific data for the upper face are not provided.^3^ According to the American Society of Plastic Surgeons, there has been a marked uptick in upper facial cosmetic surgeries during and after the COVID-19 pandemic, likely attributed to the widespread use of masks that shifted attention to the eyes and forehead.^3^ Additionally, the so-called Zoom Boom has significantly impacted the increased desire for facial aesthetic modifications, because remote work and video calls have normalized the pursuit of good appearances.^4^

Bibliometric analyses are a widely used statistical method for identifying trends in research, evaluating article performance, and describing overall patterns within a field. These analyses enable researchers to understand the current knowledge foundation, while facilitating connections with potential collaborators and uncovering novel research avenues.^5^ Although authors of bibliometric studies have previously examined the most cited articles on rhinoplasty and rhytidectomy, no comprehensive review has been conducted to assess the state of cosmetic upper facial plastic procedures.^6,7^ In this study, the authors aim to analyze the top 100 (T100) cited articles concerning cosmetic upper facial plastic procedures published from 1974 to 2024. This extensive 50-year time frame allows for a thorough investigation into the evolution of cosmetic facial surgery, potentially revealing pivotal moments in the field's history. The citation count of an article serves as a crucial metric for assessing its impact and reach within the scientific community, often correlating with factors such as the journal's impact factor (IF) and the perceived strength of the research. Furthermore, citation count analysis facilitates a balanced assessment of articles across the broader scientific community, rather than relying solely on the judgment of a single panel of reviewers. For the purposes of this study, a procedure is defined as any surgical or nonsurgical approach aimed at modifying a cosmetic outcome or feature. Throughout this study, our objective is to delineate the trends in cosmetic upper facial surgeries to enhance the understanding of the field such that clinicians, researchers, and students can identify prevailing techniques, emerging trends, and potential areas of future investigation.

## METHODS

### Search Methodology

T100 journal articles were searched and sourced from the Web of Science (WoS), a Clarivate property. The WoS, developed by Thomson Scientific, is noted as the leading academic search engine for scientific citation search.^8^

Using the WoS search engine, a search was conducted in May 2024. The total number of journals included in the WoS database for the search was 194. A comprehensive list of 21 search terms was used to effectively capture topics in cosmetic upper facial plastic research. Boolean search operators “AND” and “OR” were included between terms to incorporate as wide a search criterion as possible. The exact search terms can be found in Supplemental Table.

Search results were organized on the WoS in descending rank order, with the greatest citation counts at the top. Collected articles were categorized by a list of criteria, including citation count, title, journal of publication, corresponding author and their institution and country, publication year, article topic, and study design. Study designs included analytic studies (case control, cross sectional, prospective cohort, retrospective cohort), descriptive (case series), and interventional (clinical trials, randomized control trials, and experimental design).

### Inclusion and Exclusion Criteria

The article inclusion criteria were: (1) original reports, (2) case series, (3) case reports, (4) published between 1974 and 2024, (5) indexed in PubMed, (6) searchable by the WoS, and (7) ophthalmology or surgery journals. The article exclusion criteria were: (1) editorials, (2) reviews, (3) letters, (4) meeting abstracts, (5) proceedings papers, (6) non-English articles, and (7) nonclinical studies. All search results were assessed in a masked and duplicate fashion by 2 investigators (N.R. and E.P.). They were then cross-reviewed and evaluated by the 2 authors in a reconciliation meeting to ensure all articles met the criteria for inclusion.

### Statistical Analysis

Statistical analysis was conducted through Microsoft Excel (V16.79.1; Microsoft, Redmond, WA), including the Analysis ToolPak. A linear regression was conducted to determine trends between the year each article was published and the number of citations it received; correlation was calculated using the Excel Analysis ToolPak, and a *P*-value of <.05 was considered statistically significant.

## RESULTS

Our Supplemental Table contains the T100 cited articles in oculoplastic interventions. The table includes the rank order of the publications, the AMA citation, and the corresponding number of citations each article has garnered. The rank 100 article was distinguished as the most recently published article from other relevant articles with the same citation count.

Through a statistical analysis of the number of citations received in each article in the T100, the authors reveal an average of 55.1 citations (a standard deviation of 38.7), a median of 40, and a mode of 30. The citations received ranged from 218 to 29.

**Figure 1.**
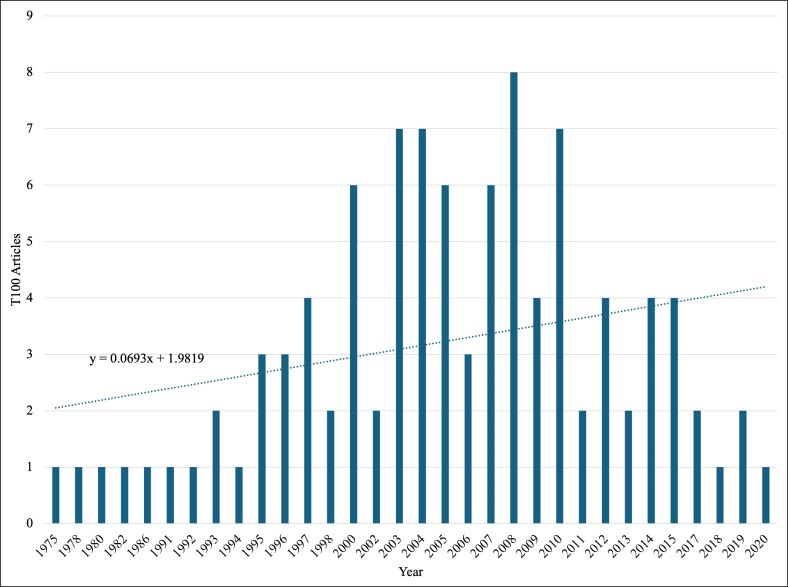
Distribution of the top 100 (T100) articles by year of publication. A histogram of the frequency of T100 articles found in the years between 1975 and 2020. Overlaying the histogram is a trend line from which we can see the positive correlation between the year and the amount of T100 publications included.

From 1974 to 2024, there was a weak positive correlation between increasing year of publication and inclusion of the article in the T100 (0.3497, *R*^2^ = 0.1223, *P* < .05; Figure 1). The year 2008 saw the most publications included at 8% of the T100. Figure 1 exhibits a negative skew in the distribution of T100 publications by year. The decade between 2000 and 2010 saw the most publications on the list.

**Figure 2.**
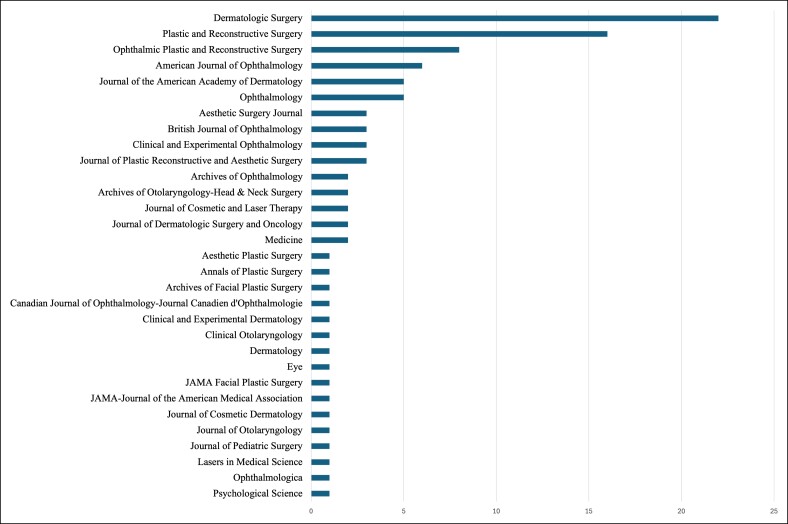
Distribution of top 100 (T100) articles by journal of publication. A bar chart arranged in order of decreasing total amounts of T100 articles included for each journal of publication we found. The *y* axis lists each unique journal included in the T100 list of publications, and the *x* axis contains the total number of T100 publications for that journal.

Thirty-one unique journals were represented in the T100 articles in upper facial plastic surgery (Figure 2). *Dermatologic Surgery*, *Plastic and Reconstructive Surgery*, and *Ophthalmic Plastic and Reconstructive Surgery* were the 3 most represented journals, with 22%, 16%, and 8% of the T100 articles, respectively. The Scimago Journal Ranking (SJR) H-indexes of these journals are 141, 210, and 69, respectively. The 2022 journal IF is 2.4, 3.6, and 2.0, respectively. Although SJR utilizes its proprietary PageRank algorithm to estimate the journal “prestige,” both SJR and IF values serve as established methods of determining the level of impact for journals.^9^ Sixteen journals were represented by a single publication in the T100 articles.

Table 1 lists each journal and its associated 2022 IF and 5-year average IF taken from the 2022 WoS report. A regression of the IF and papers included in the T100 list showed that the 2022 IF was not significant in determining the likelihood of inclusion in the T100 (corr = 9.85%, *R*^2^ = 0.0097, *P* = .58). A similar regression on the 5-year average journal IF and inclusion in the T100 also revealed that this variable was not a significant predictor for inclusion (corr = 5.29%, *R*^2^ = 0.0028, *P* = .78). Lastly, a multivariate regression of 2022 IF and 5-year average journal IF with the number of papers included in the T100 determined that neither variable was a statistically significant predictor of inclusion in the T100 (2022 IF *P* = .66, 5-year IF *P* = .98, *R*^2^ = 0.0097).

**Table 1.** Journal and its Associated 2022 Impact Factor and 5-Year Average Impact Factor Taken From the 2022 Web of Science Report

Journal	2022 Impact factor	5-Year impact factor
*Psychological Science*	1.90	2.70
*Ophthalmologica*	3.60	4.20
*Lasers in Medical Science*	4.20	4.90
*Journal of Pediatric Surgery*	2.60	2.70
*Journal of Otolaryngology*	13.80	11.10
*Journal of Cosmetic Dermatology*	13.70	12.80
*JAMA—Journal of the American Medical Association*	2.70	2.90
*JAMA Facial Plastic Surgery*	2.33	2.31
*Eye*	3.10	3.50
*Dermatology*	1.90	2.10
*Clinical Otolaryngology*	23.00	2.50
*Clinical and Experimental Dermatology*	1.20	1.60
*Canadian Journal of Ophthalmology–Journal Canadien d’Ophthalmologie*	3.90	4.00
*Archives of Facial Plastic Surgery*	2.40	2.40
*Annals of Plastic Surgery*	8.20	8.40
*Aesthetic Plastic Surgery*	3.40	3.30
*Medicine*	2.60	2.70
*Journal of Dermatologic Surgery and Oncology*	2.40	2.70
*Journal of Cosmetic and Laser Therapy*	2.10	2.50
*Archives of Otolaryngology–Head and Neck Surgery*	2.40	2.60
*Archives of Ophthalmology*	2.40	2.70
*Journal of Plastic Reconstructive and Aesthetic Surgery*	4.67	4.26
*Clinical and Experimental Ophthalmology*	2.90	2.90
*British Journal of Ophthalmology*	3.40	3.80
*Aesthetic Surgery Journal*	4.00	4.10
*Ophthalmology*	4.10	3.40
*Journal of the American Academy of Dermatology*	4.20	2.60
*American Journal of Ophthalmology*	1.29	1.48
*Ophthalmic Plastic and Reconstructive Surgery*	1.80	1.70
*Plastic and Reconstructive Surgery*	4.10	4.30
*Dermatologic Surgery*	4.34	4.37

**Figure 3.**
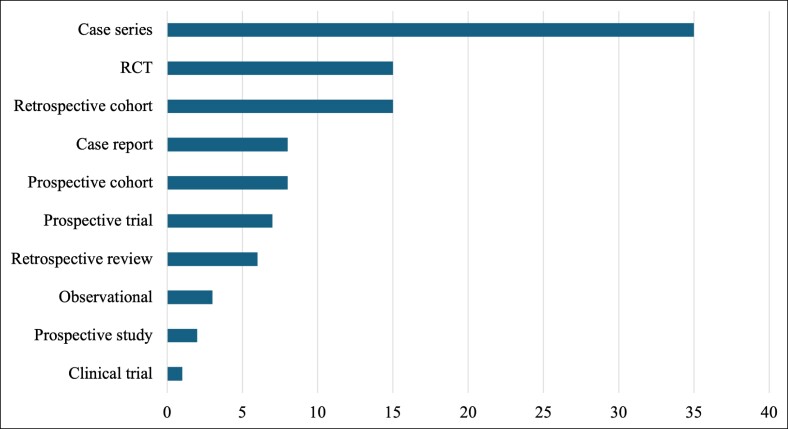
Distribution of top 100 (T100) articles by study design. A bar chart that depicts the number of papers that followed 1 of 10 unique study designs that were included in the T100 list. The results are in descending order of the number of papers per study design.

The papers that consist of the T100 articles encompass 10 distinct types of study designs in their investigations. The most common among the T100 was a case series report at 35% of the T100 articles (Figure 3). A retrospective cohort study (15%) and a randomized control trial (RCT; 15%) were the second most common study designs.

**Figure 4.**
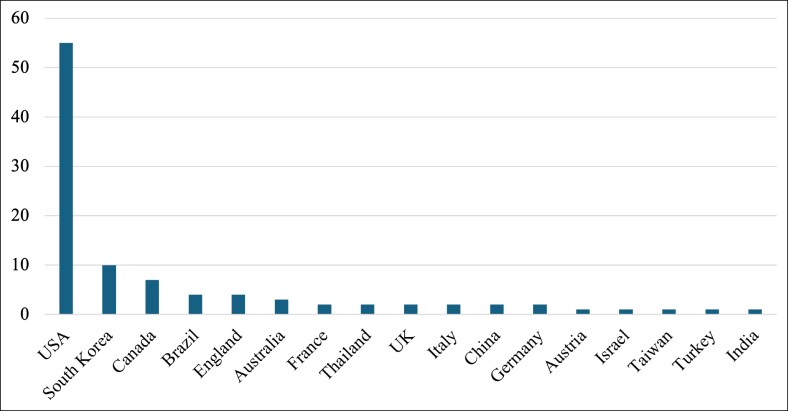
Distribution of top 100 (T100) articles by country. This figure is a horizontal bar chart that depicts the number of publications for each unique country of publication that we included in the T100 list. The left-most country included the most papers, whereas the right represents the country that accounts for fewer papers.

In a breakdown on articles by country by correspondence address, a total of 17 countries were represented in the WoS. For the purposes of this study, articles that listed addresses in England and Scotland were classified as the United Kingdom. Articles from the United States comprised 55% of the T100 publications, with South Korea (10%) being the second most represented and Canada (7%) the third (Figure 4).

**Figure 5.**
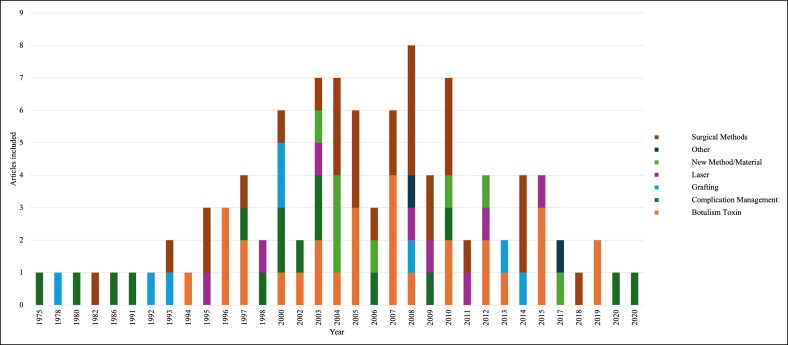
Top 100 (T100) article area of study by year. This figure is similar to Figure 1 in the sense that it is a histogram depicting the frequency of T100 papers over the 50-year time period that we studied. It includes further detail in that it depicts the frequency of area of study in each year there is an article. The different areas of study are color coded and clarified by the legend on the right margin of the figure. Areas of study include surgical methods, new methods/materials, laser, grafting, complication management, botulinum toxin, and other.

The T100 articles were classified based on 7 general study areas depicted in Table 2. Surgical methods were the most common unique study area, with 30% of the T100. The second and third most common unique study areas were botulinum toxin and complication management, with 29% and 15% of the T100, respectively. The unique study area with the highest average citations received was botulinum toxin with an average of 64.7 citations (standard deviation = 48.4). Figure 5 depicts the representation of topics throughout each year a T100 article was published.

**Table 2.** Classification of Top 100 Articles Based on 7 General Study Areas

Unique study area	No. of articles	Average citation	Standard deviation
Surgical methods	30	49.8	25.3
Botox	29	64.7	48.4
Complication management	15	38.2	18.6
Laser	8	63.4	58.1
New methods/materials	8	56.8	40.0
Grafting	8	64.9	32.2
Other	2	44	3

**Figure 6.**
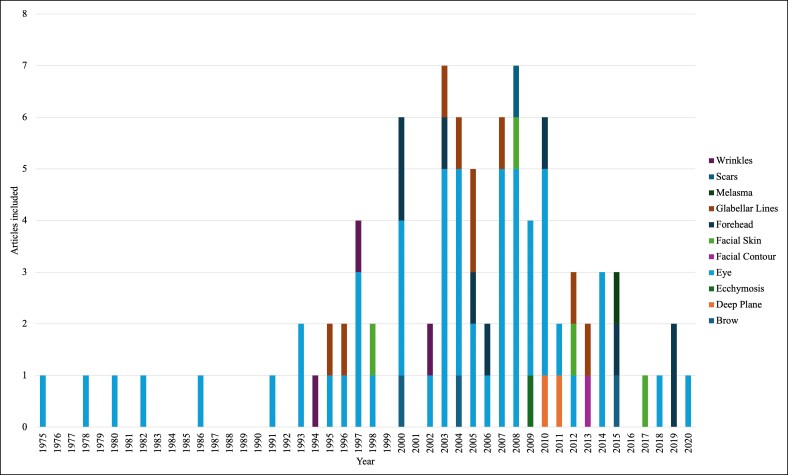
Top 100 (T100) target structure by year constructed with a similar methodology as Figure 5 but instead shows details about which suture the publications in upper facial plastic surgery are referencing. This figure is a histogram that shows T100 publications by year with a color-coded legend on the right margin corresponding to the colors in the histogram bars in the graph. The structures specified are wrinkles, scars, melasma, glabellar lines, forehead, facial skin, facial contour, eye, ecchymosis, deep plane, and brow.

The T100 articles were also separated into groups based on the anatomical structure that was the focus of the article (Table 3). Twelve unique structures were identified, with a specific anatomic structure not identified in 12 articles. The eye was the most common structure of focus, with 54% of the T100 articles, followed by glabellar lines (9%) and the forehead (9%). The structure of focus that was covered by >1 article that received the highest average citations was glabellar lines, with an average of 69.0 citations (standard deviation = 37.9). The articles in which a structure of focus was not specified received an average of 103.0 citations (standard deviation = 65.1). Figure 6 depicts the representation of target structures throughout each year a T100 article was published.

**Table 3.** Top 100 Articles Were Separated into Groups Based on the Frequency of Anatomical Target Structures

Unique structure targeted	No. of articles	Average citation	Standard deviation
Eye	54	42.3	18.9
Glabellar lines	9	69.0	37.9
Forehead	9	61.3	50.5
Facial skin	4	49.0	8.8
Wrinkles	3	63.3	55.2
Brow	3	60.0	26.0
Deep plane	2	39.0	9.9
Scars	1	54.0	NA
Facial contour	1	50.0	NA
Melasma	1	34.0	NA
Ecchymosis	1	34.0	NA

NA, not applicable.

**Figure 7.**
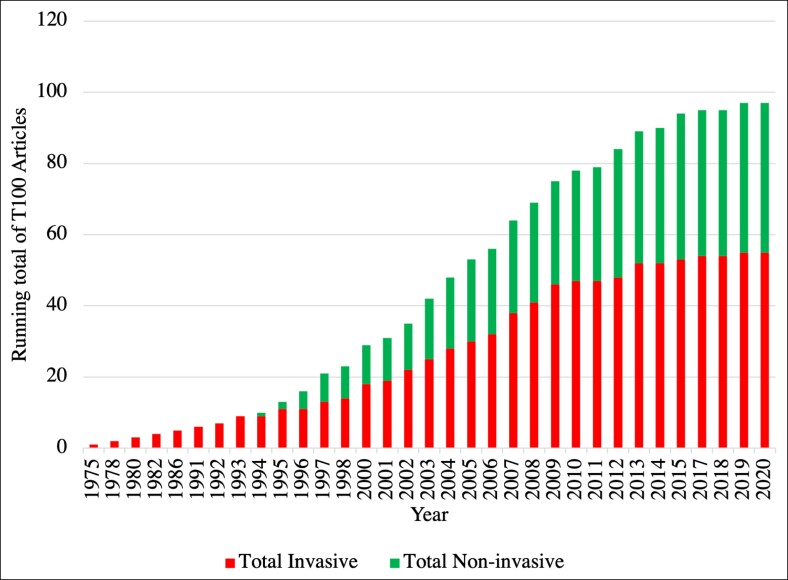
Top 100 (T100) focus on invasive procedures vs noninvasive procedures by year. Depicts the increasing running total of T100 publications over the 50-year period of study. Each successive year is a running total of all published T100 articles ending with 99 articles in 2020 (1 article was excluded in this graph). The articles are then classified into invasive or noninvasive categories based on the procedures discussed by the authors. Articles covering invasive topics are represented in the lower bars while noninvase articles are represented above the line demarcation where applicable from years 1994 to 2020. This figure shows the rising proportion of T100 articles that cover noninvasive topics further and further into the study period.

Articles were categorized based on whether or not the interventions discussed were invasive or noninvasive procedures. Three articles were not able to be categorized on this criterion. Invasive procedures made up 55% of the T100 articles, whereas noninvasive procedures were 44% of the T100 articles. One article did not fit either category. Figure 7 depicts the distribution of invasive vs noninvasive articles across the 50-year period of study.

## DISCUSSION

In this study, we conducted a comprehensive analysis of the T100 cited articles in cosmetic upper facial plastic surgery. To the best of our knowledge, we are the first to specifically investigate multidisciplinary academic research focused on procedures involving the upper face, which includes the eye and orbit, forehead, and temporal region. Interest in cosmetic plastic surgery research has broadly grown over the past 2 decades.^9,10^ The authors of previous research have shown increased interest in facial cosmetic surgery, driven by heightened visual scrutiny associated with popular media. This interest has been further intensified by the hyperfocus on the upper face during the pandemic era.^8,11^

Our findings align with similar bibliometric analyses, revealing that the majority of highly cited articles originate from US-based centers. Interestingly, publication trends are more skewed toward reconstructive plastic surgery papers compared with cosmetic ones.^12^ This trend mirrors previous bibliometric analyses in ophthalmology.^13,14^ Importantly, our search criteria excluded non-English language papers, which may have further shifted the count of included articles toward the United States. This is particularly relevant because 18 of the 20 countries represented in the T100 have a non-English national language. Reviewer bias favoring articles from the United States and other high-resource, high-income countries, including South Korea, has been previously identified.^15,16^ East Asian countries, in particular, have a growing demand for cosmetic surgeries, especially oculofacial procedures such as blepharoplasty.^17^ This interest may explain the greater proportion of East Asian–origin oculoplastic research compared with other bibliometric analyses, which tend to favor Western-origin papers.^18^

In our analysis of the T100 articles related to upper facial plastic surgery interventions, we found that discussions of surgical methods were the most published topic, with 30 articles (Table 2). In other bibliometric analyses of facial plastic surgery trends, we revealed a similar emphasis on specific surgical techniques, with facial rejuvenation surgery being the most focused operation.^19-21^ Because in our bibliometric analysis we focused on upper facial cosmetic plastic surgery procedures, some of the surgical methods discussed in highly cited papers included operations such as endoscopic brow lift, blepharoplasty, forehead contouring, and transblepharoplasty forehead lift.^22-24^ In bibliometric analyses adjacent to this study, authors have noted a significant shift in patient interest for facial cosmetic surgeries after the initial March 2020 onset of the COVID-19 pandemic.^25^ Although in our findings we do not report a significant shift in research citations in the years after 2020 because of the lengthy period of the publications that we analyzed, in these findings we concur with the fact that these procedures are increasing in popularity within the research space and the patient population.

Botulinum toxin was the second most represented topic in the T100 articles, with 29 articles (Table 2). For the purposes of the study, botulinum toxin usage was analyzed only in relation to its cosmetic applications and categorized as minimally/noninvasive. Botox is a useful tool for treating glabellar lines, crow's feet, and forehead wrinkles.^26^ The most cited article in the T100, authored by Cole et al, was a comprehensive review of all adverse events reported to the Federal Drug Administration related to the use of botulinum toxin. Although Botox injections have been in use since the 1970s, their cosmetic applications, particularly for glabellar line minification, have increased in the last 30 years following a landmark clinical trial in 1994.^27^ This paper is pivotal because of its substantial dataset and the generalizability of its conclusions. Moreover, of the 29 botulinum toxin articles in the T100 over 50 years, 11 were published in the 10 years following 1994. Dynamic wrinkles are the primary cosmetic indication for Botox injections and are among the most common cosmetic procedures performed worldwide.^28^ This is further evidenced by 14 out of 29 botulinum articles in the T100 directly focusing on wrinkle reduction.

Increased acceptance and interest in cosmetic and noninvasive procedures have driven the growth of more skin enhancement techniques such as CO_2_ laser resurfacing. In the second most highly cited article, Waldorf et al report the results of laser treatments on periorbital, glabellar, or perioral rhytides. In this paper, the authors followed 47 patients to evaluate the effectiveness of the procedure, reporting improvements and adverse effects. Although it is the second highest cited paper overall, it is the most cited paper in which the authors focused on laser resurfacing techniques. CO_2_ laser therapy was previously the only laser skin rejuvenation technique available, which may explain the relatively high citation count of this article compared with non-CO_2_ laser-themed papers in the T100.^29^ However, of the 8 articles in the T100 related to lasers, 6 primarily centered around either erbium:YAG or Nd:YAG laser technology. These lasers, in contrast to CO_2_, are notable for greater efficacy in more superficial skin indications, signaling an overall growing interest in less invasive yet highly effective cosmetic enhancement procedures among the public.^30,31^

The authors of other reviews on plastic surgery have observed a similar growing interest in minimally invasive or noninvasive procedures, with research in this area gaining momentum over time.^32,33^ In our review, we numerically demonstrate that researchers are increasingly seeking improved techniques to address cosmetic concerns through minimally invasive methods. This trend highlights a growing recognition of the importance of effective, less invasive solutions in cosmetic surgery, reflecting evolving patient preferences and a broader shift in clinical practice. This evolution in the field may continue to have implications in the field, especially in regard to residency training. In analyses of graduating plastic surgery residents’ perceptions of their training in minimally invasive and cosmetic surgery, authors have demonstrated an increase of perceptions over time, likely reflecting increased emphasis in training commensurate with increased demand.^34-36^

Table 3 outlines the frequency of anatomical target structures represented in the T100 articles. Notably, the authors of most articles focused on the periorbital region and interventions related to the cosmetic enhancement of the eye. In studies in which authors are tracking trends in cosmetic interventions for the eye, a high degree of interest in this research area is revealed.^37^ Additionally, researchers assessing patient interest in specific procedures indicate that blepharoplasty and brow lift are the most requested by patients.^38^ Interestingly, 12% of the T100 papers were not specific to any one region of the upper face, reflecting a broader range of research objectives. Researchers concentrating on specific anatomical structures, such as the nose, eyes, or forehead, in their articles tend to explore specialized techniques and outcomes in greater depth.^37^ In the significant proportion of papers included in our search, authors corroborate similar trends, demonstrating that those discussing combined approaches to surgical techniques often achieve higher citation counts in their studies.^39^

Because blepharoplasty and eye-related procedures were prominently represented in our T100 search of upper facial plastic surgery, this procedure offers a clear view of its evolution over the past 50 years. In earlier articles, such as those by Smith et al (Rank 14) and Harley et al (Rank 65), the authors primarily focused on complication management, reflecting the older techniques that involved excising skin, muscle, and fat, which sometimes resulted in paradoxical cosmetic defects.^40^ In contrast, in more recent articles regarding periorbital procedures, authors indicate a shift toward the transconjunctival approach for lower lid blepharoplasty, as seen in Ghabrial et al (Rank 85). Additionally, there has been an increased use of lasers in eyelid and upper facial rejuvenation, highlighted in studies by Tierney et al (Rank 47). These advancements support a trend that began in the late 1990s with the introduction of these technologies and methods.^41^ Overall, in the T100 articles, the authors illustrate a trend in which authors of newer publications increasingly focus on innovative technologies and more conservative techniques, enhancing and refining the methods and materials used in earlier studies.

Of the 15 distinct study designs categorized from the T100 articles, case series emerged as the most predominant at 35%, followed by retrospective cohort studies at 15% and RCTs at 15%. Although RCTs are widely recognized as the gold standard in clinical research, they are often challenging to implement in surgical contexts.^42^ Difficulties in standardizing RCTs for surgical interventions include the challenges of creating blinded designs for surgeons and a general lack of funding and infrastructure for surgical technique-based RCTs.^43^ Given that in our research we demonstrated a focus on oculoplastic interventions, it was expected that case series and retrospective cohort reviews would comprise the majority of study designs. Case series, in particular, have a long-standing tradition in surgical literature.^44^ This trend highlights the ongoing reliance on these study designs to inform practice in cosmetic upper facial plastics.

Throughout the 50-year analysis of upper facial plastic surgery articles, a weak positive correlation was identified between the progressive year of publication and inclusion in the T100 articles, with a correlation coefficient of 0.3497, *R*^2^ = 0.1223, *P* < .05. This finding is significant, because authors of previous studies have frequently pointed out that the citation time window poses a major limitation in bibliometric analyses.^42,43,45,46^ The results indicate that the authors in their analysis do not exclusively favor older published articles; however, it is important to note that the lack of time available for articles published in the last 4 years to accumulate citations does influence their representation. Of the 32 unique years represented in the analysis, 90% of the articles were published between 1995 and 2010, indicating a substantial increase compared with earlier decades. Understanding this surge in highly cited publications is multifactorial.

Firstly, research productivity has dramatically increased among physicians, particularly in surgical subspecialties, because research output has become increasingly valuable for securing positions in highly competitive specialties.^47^ In the field of plastic surgery, the average number of citations per surgeon has increased significantly, from 5 in the 1980s to 7 in the 1990s, and further to 8 in the 2010s (*P* < .01).^48^ Moreover, interest in cosmetic plastic surgery has expanded over several decades. The 1990s demonstrated a 725% increase in cosmetic surgery procedures from 1992 to 2005, corresponding to 10 billion dollars in overall spending.^49^ Importantly, it is noted that facial plastic procedures were a major driving force in this boom, suggesting the beginning of a trend that continues today.^50^ This evolving landscape has contributed to the proliferation of research and the rising prominence of upper facial plastic surgery in academic literature, reflecting broader societal trends and shifts in patient demand.

There are several potential limitations to the data collected in this bibliometric study. Firstly, the search was conducted solely within the WoS database. Including additional databases, such as Scopus or Ovid, may have yielded different results, suggesting that in future studies, researchers should incorporate multiple databases for a more comprehensive data collection exercise. Secondly, our analysis was limited to articles published between May 1, 1974, and May 1, 2024, to visualize overarching trends in publications. However, this 50-year time frame may have been too broad to effectively target specific topics, such as the use of laser cosmetic surgery, which is a relatively recent development. In previous citation analyses, authors indicate that researchers focus on emerging techniques typically require up to 2 years to begin accumulating citations in their papers, and it can take up to 10 years for them to reach a stable peak citation count.^51^ Consequently, within our 50-year scope, some articles may have become outdated as newer interventions rapidly emerged.

In a significant portion of our bibliometric analysis, we relied on 21 carefully selected search terms designed to encompass a wide range of topics within upper facial plastic interventions, while excluding irrelevant articles. These terms generated over 16,000 publications, necessitating a manual review to ensure relevance to the study's goals. This large volume of results required a masked and duplicate review process, in which researchers individually assessed the T100 cited articles related to oculoplastic interventions before reconciling a final list. Despite this thorough approach, there remains a risk of human error in data extraction, which may have affected the study's specificity. In future studies, benefit might be had from adjusting their review processes or increasing the number of researchers involved to enhance the accuracy of article selection.

## CONCLUSIONS

In this comprehensive bibliometric analysis, the authors examine the T100 most cited articles on cosmetic upper facial plastic surgery over the past 5 decades. They highlight significant innovations in surgical techniques and a growing emphasis on minimally invasive procedures, particularly focusing on the use of botulinum toxin and laser technology. Notably, there was a marked growth in publications during the late 1990s and early 2000s, reflecting increased research activity and advancements in the field during that period. In this study, the authors also underscore the importance of these pioneering works in shaping current practices and guiding future research in cosmetic upper facial plastic surgery. This growing body of literature continues to inform and enhance clinical outcomes, ensuring that patients benefit from the latest scientific and technological developments.

## Supplementary Material

ojae123_Supplementary_Data
