# Supplementary material for: Bibliometric Analysis of the Highest Cited Cosmetic Upper Facial Plastic Surgery Articles Over 50 Years
Source: Aesthet Surg J Open Forum. 2024 Dec 16;7:ojae123. doi: 10.1093/asjof/ojae123 (PMC11758881; doi:10.1093/asjof/ojae123)
Supplement: ojae123_Supplementary_Data [file ojae123_supplementary_data.docx]

Supplemental Table: The search terms used were: (“cosmetic*” AND “bleph*”) OR (“cosmetic*” AND “upper eyelid blepharoplasty”) OR (“cosmetic*” AND “lower eyelid blepharoplasty”) OR (“cosmetic*” AND “orbital fat transposition”) OR (“cosmetic*” AND “orbital fat reduction”) OR (“cosmetic*” AND “fat graft”) OR (“cosmetic*” AND “orbicularis sling”) OR (“cosmetic*” AND “lateral canthoplasty”) OR (“cosmetic*” AND “ptosis repair”) OR (“cosmetic*” AND “forehead lift”) OR (“cosmetic*” AND “brow lift”) OR (“cosmetic*” AND “endoscopic brow lift”) OR (“cosmetic*” AND “pretrichial brow lift”) OR (“cosmetic*” AND “trichophytic brow lift”) OR (“cosmetic*” AND “thread lift”) OR (“cosmetic*” AND “coronal brow lift”) OR (“cosmetic*” AND “eyelid fillers”) OR (“cosmetic*” AND “botulinum toxin”) OR (“cosmetic*” AND “chemical peel”) OR (“cosmetic*” AND “laser resurfacing”) OR (“cosmetic*” AND “retraction repair”). The following table contains the publications which were included in our T100 list of most cited articles.

| **Rank** | **Article citation** | **Number of citations** |
| --- | --- | --- |
| 1 | Coté TR, Mohan AK, Polder JA, Walton MK, Braun MM. Botulinum toxin type A injections: adverse events reported to the US Food and Drug Administration in therapeutic and cosmetic cases. J Am Acad Dermatol. 2005;53(3):407-415. doi:10.1016/j.jaad.2005.06.011 | 218 |
| 2 | Waldorf HA, Kauvar AN, Geronemus RG. Skin resurfacing of fine to deep rhytides using a char-free carbon dioxide laser in 47 patients. Dermatol Surg. 1995;21(11):940-946. doi:10.1111/j.1524-4725.1995.tb00530.x | 215 |
| 3 | Alam M, White LE, Martin N, Witherspoon J, Yoo S, West DP. Ultrasound tightening of facial and neck skin: a rater-blinded prospective cohort study. J Am Acad Dermatol. 2010;62(2):262-269. doi:10.1016/j.jaad.2009.06.039 | 159 |
| 4 | Havas DA, Glenberg AM, Gutowski KA, Lucarelli MJ, Davidson RJ. Cosmetic use of botulinum toxin-a affects processing of emotional language. Psychol Sci. 2010;21(7):895-900. doi:10.1177/0956797610374742 | 155 |
| 5 | Garcia A, Fulton JE Jr. Cosmetic denervation of the muscles of facial expression with botulinum toxin. A dose-response study. Dermatol Surg. 1996;22(1):39-43. doi:10.1111/j.1524-4725.1996.tb00569.x | 141 |
| 6 | Ascher B, Zakine B, Kestemont P, Baspeyras M, Bougara A, Santini J. A multicenter, randomized, double-blind, placebo-controlled study of efficacy and safety of 3 doses of botulinum toxin A in the treatment of glabellar lines [published correction appears in J Am Acad Dermatol. 2005 Jan;52(1):156]. J Am Acad Dermatol. 2004;51(2):223-233. doi:10.1016/j.jaad.2003.11.084 | 134 |
| 7 | Pinski KS, Roenigk HH Jr. Autologous fat transplantation. Long-term follow-up. J Dermatol Surg Oncol. 1992;18(3):179-184. doi:10.1111/j.1524-4725.1992.tb02795.x | 133 |
| 8 | Ben Simon GJ, Macedo AA, Schwarcz RM, Wang DY, McCann JD, Goldberg RA. Frontalis suspension for upper eyelid ptosis: evaluation of different surgical designs and suture material. Am J Ophthalmol. 2005;140(5):877-885. doi:10.1016/j.ajo.2005.05.031 | 127 |
| 9 | Keen M, Blitzer A, Aviv J, et al. Botulinum toxin A for hyperkinetic facial lines: results of a double-blind, placebo-controlled study. Plast Reconstr Surg. 1994;94(1):94-99. doi:10.1097/00006534-199407000-00009 | 127 |
| 10 | Carruthers A, Carruthers J. Prospective, double-blind, randomized, parallel-group, dose-ranging study of botulinum toxin type A in men with glabellar rhytids. Dermatol Surg. 2005;31(10):1297-1303. doi:10.1111/j.1524-4725.2005.31206 | 121 |
| 11 | Ben Simon GJ, Lee S, Schwarcz RM, McCann JD, Goldberg RA. External levator advancement vs Müller's muscle-conjunctival resection for correction of upper eyelid involutional ptosis. Am J Ophthalmol. 2005;140(3):426-432. doi:10.1016/j.ajo.2005.03.033 | 109 |
| 12 | Chertow DS, Tan ET, Maslanka SE, et al. Botulism in 4 adults following cosmetic injections with an unlicensed, highly concentrated botulinum preparation. JAMA. 2006;296(20):2476-2479. doi:10.1001/jama.296.20.2476 | 97 |
| 13 | Hass AN, Penne RB, Stefanyszyn MA, Flanagan JC. Incidence of postblepharoplasty orbital hemorrhage and associated visual loss [published correction appears in Ophthal Plast Reconstr Surg. 2005 Mar;21(2):169]. Ophthalmic Plast Reconstr Surg. 2004;20(6):426-432. doi:10.1097/01.iop.0000143711.48389.c5 | 94 |
| 14 | Smith B, Petrelli R. Dermis-fat graft as a movable implant within the muscle cone. Am J Ophthalmol. 1978;85(1):62-66. doi:10.1016/s0002-9394(14)76666-8 | 92 |
| 15 | Carruthers A, Carruthers J, Cohen J. A prospective, double-blind, randomized, parallel- group, dose-ranging study of botulinum toxin type a in female subjects with horizontal forehead rhytides. Dermatol Surg. 2003;29(5):461-467. doi:10.1046/j.1524-4725.2003.29114.x | 86 |
| 16 | Hexsel DM, De Almeida AT, Rutowitsch M, et al. Multicenter, double-blind study of the efficacy of injections with botulinum toxin type A reconstituted up to six consecutive weeks before application. Dermatol Surg. 2003;29(5):523-529. doi:10.1046/j.1524-4725.2003.29121.x | 82 |
| 17 | Ahn MS, Catten M, Maas CS. Temporal brow lift using botulinum toxin A. Plast Reconstr Surg. 2000;105(3):1129-1139. doi:10.1097/00006534-200003000-00046 | 76 |
| 18 | Booth AJ, Murray A, Tyers AG. The direct brow lift: efficacy, complications, and patient satisfaction. Br J Ophthalmol. 2004;88(5):688-691. doi:10.1136/bjo.2003.019232 | 74 |
| 19 | Knize DM. Transpalpebral approach to the corrugator supercilii and procerus muscles. Plast Reconstr Surg. 1995;95(1):52-62. | 72 |
| 20 | Lee MJ, Oh JY, Choung HK, Kim NJ, Sung MS, Khwarg SI. Frontalis sling operation using silicone rod compared with preserved fascia lata for congenital ptosis a three-year follow-up study. Ophthalmology. 2009;116(1):123-129. doi:10.1016/j.ophtha.2008.08.049 | 69 |
| 21 | Yoon JS, Lee SY. Long-term functional and cosmetic outcomes after frontalis suspension using autogenous fascia lata for pediatric congenital ptosis. Ophthalmology. 2009;116(7):1405-1414. doi:10.1016/j.ophtha.2009.01.040 | 67 |
| 22 | Steinkogler FJ, Kuchar A, Huber E, Arocker-Mettinger E. Gore-Tex soft-tissue patch frontalis suspension technique in congenital ptosis and in blepharophimosis-ptosis syndrome. Plast Reconstr Surg. 1993;92(6):1057-1060. doi:10.1097/00006534-199311000-00009 | 67 |
| 23 | Gerth DJ, King B, Rabach L, Glasgold RA, Glasgold MJ. Long-term volumetric retention of autologous fat grafting processed with closed-membrane filtration. Aesthet Surg J. 2014;34(7):985-994. doi:10.1177/1090820X14542649 | 65 |
| 24 | Ablon G, Rotunda AM. Treatment of lower eyelid fat pads using phosphatidylcholine: clinical trial and review. Dermatol Surg. 2004;30(3):422-428. doi:10.1111/j.1524-4725.2004.30114.x | 65 |
| 25 | McGraw BL, Adamson PA. Postblepharoplasty ectropion. Prevention and management. Arch Otolaryngol Head Neck Surg. 1991;117(8):852-856. doi:10.1001/archotol.1991.01870200046006 | 65 |
| 26 | Fagien S, Cox SE, Finn JC, Werschler WP, Kowalski JW. Patient-reported outcomes with botulinum toxin type A treatment of glabellar rhytids: a double-blind, randomized, placebo-controlled study. Dermatol Surg. 2007;33(1 Spec No.):S2-S9. doi:10.1111/j.1524-4725.2006.32325.x | 64 |
| 27 | Dresner SC. Further modifications of the Müller's muscle-conjunctival resection procedure for blepharoptosis. Ophthalmic Plast Reconstr Surg. 1991;7(2):114-122. doi:10.1097/00002341-199106000-00005 | 63 |
| 28 | Polnikorn N. Treatment of refractory dermal melasma with the MedLite C6 Q-switched Nd:YAG laser: two case reports. J Cosmet Laser Ther. 2008;10(3):167-173. doi:10.1080/14764170802179687 | 62 |
| 29 | Patel MP, Shapiro MD, Spinelli HM. Combined hard palate spacer graft, midface suspension, and lateral canthoplasty for lower eyelid retraction: a tripartite approach. Plast Reconstr Surg. 2005 Jun;115(7):2105-14; discussion 2115-7. doi: 10.1097/01.prs.0000164677.25488.49. PMID: 15923862. | 60 |
| 30 | Glat PM, Jelks GW, Jelks EB, Wood M, Gadangi P, Longaker MT. Evolution of the lateral canthoplasty: techniques and indications. Plast Reconstr Surg. 1997 Nov;100(6):1396-405; discussion 1406-8. doi: 10.1097/00006534-199711000-00003. PMID: 9385950. | 58 |
| 31 | Hexsel D, Dal'Forno T, Hexsel C, Do Prado DZ, Lima MM. A randomized pilot study comparing the action halos of two commercial preparations of botulinum toxin type A. Dermatol Surg. 2008 Jan;34(1):52-9. doi: 10.1111/j.1524-4725.2007.34008.x. Epub 2007 Dec 5. PMID: 18053050. | 56 |
| 32 | Elner VM, Hassan AS, Frueh BR. Graded full-thickness anterior blepharotomy for upper eyelid retraction. Trans Am Ophthalmol Soc. 2003;101:67-73; discussion 73-5. PMID: 14971565; PMCID: PMC1358976. | 56 |
| 33 | Armstrong MW, Mountain RE, Murray JA. Treatment of facial synkinesis and facial asymmetry with botulinum toxin type A following facial nerve palsy. Clin Otolaryngol Allied Sci. 1996 Feb;21(1):15-20. doi: 10.1111/j.1365-2273.1996.tb01018.x. PMID: 8674216. | 55 |
| 34 | Caviggioli F, Klinger F, Villani F, Fossati C, Vinci V, Klinger M. Correction of cicatricial ectropion by autologous fat graft. Aesthetic Plast Surg. 2008 May;32(3):555-7. doi: 10.1007/s00266-008-9117-y. PMID: 18293030. | 54 |
| 35 | Li J, Gao J, Cha P, Chang Q, Liao Y, Liu C, Li K, Lu F. Supplementing fat grafts with adipose stromal cells for cosmetic facial contouring. Dermatol Surg. 2013 Mar;39(3 Pt 1):449-56. doi: 10.1111/dsu.12058. Epub 2012 Dec 28. PMID: 23279651. | 50 |
| 36 | Hwang HS, Spiegel JH. The effect of "single" vs "double" eyelids on the perceived attractiveness of Chinese women. Aesthet Surg J. 2014 Mar;34(3):374-82. doi: 10.1177/1090820X14523020. Epub 2014 Mar 6. PMID: 24604790. | 49 |
| 37 | Hexsel D, Brum C, Siega C, Schilling-Souza J, Dal'Forno T, Heckmann M, Rodrigues TC. Evaluation of self-esteem and depression symptoms in depressed and nondepressed subjects treated with onabotulinumtoxinA for glabellar lines. Dermatol Surg. 2013 Jul;39(7):1088-96. doi: 10.1111/dsu.12175. Epub 2013 Mar 6. PMID: 23465042. | 49 |
| 38 | Suh DH, Oh YJ, Lee SJ, Rho JH, Song KY, Kim NI, Shin MK. A intense-focused ultrasound tightening for the treatment of infraorbital laxity. J Cosmet Laser Ther. 2012 Dec;14(6):290-5. doi: 10.3109/14764172.2012.738912. PMID: 23057597. | 48 |
| 39 | Weinfeld AB, Burke R, Codner MA. The comprehensive management of chemosis following cosmetic lower blepharoplasty. Plast Reconstr Surg. 2008 Aug;122(2):579-586. doi: 10.1097/PRS.0b013e31818001d0. PMID: 18626377. | 47 |
| 40 | Swanson E. Objective assessment of change in apparent age after facial rejuvenation surgery. J Plast Reconstr Aesthet Surg. 2011 Sep;64(9):1124-31. doi: 10.1016/j.bjps.2011.04.004. Epub 2011 Apr 22. PMID: 21514913. | 46 |
| 41 | Weinstein C. Computerized scanning erbium:YAG laser for skin resurfacing. Dermatol Surg. 1998 Jan;24(1):83-9. doi: 10.1111/j.1524-4725.1998.tb04058.x. PMID: 9464295. | 46 |
| 42 | Cervelli V, Nicoli F, Spallone D, Verardi S, Sorge R, Nicoli M, Balzani A. Treatment of traumatic scars using fat grafts mixed with platelet-rich plasma, and resurfacing of skin with the 1540 nm nonablative laser. Clin Exp Dermatol. 2012 Jan;37(1):55-61. doi: 10.1111/j.1365-2230.2011.04199.x. PMID: 22182435. | 45 |
| 43 | Guyuron B, Michelow BJ, Thomas T. Corrugator supercilii muscle resection through blepharoplasty incision. Plast Reconstr Surg. 1995 Apr;95(4):691-6. doi: 10.1097/00006534-199504000-00010. PMID: 7892313. | 45 |
| 44 | Kang SH, Byun EJ, Kim HS. Vertical Lifting: A New Optimal Thread Lifting Technique for Asians. Dermatol Surg. 2017 Oct;43(10):1263-1270. doi: 10.1097/DSS.0000000000001169. PMID: 28430736. | 43 |
| 45 | Putterman AM. Temporary blindness after cosmetic blepharoplasty. Am J Ophthalmol. 1975 Dec;80(6):1081-3. doi: 10.1016/0002-9394(75)90339-6. PMID: 1200102. | 43 |
| 46 | Cotofana S, Freytag DL, Frank K, Sattler S, Landau M, Pavicic T, Fabi S, Lachman N, Hernandez CA, Green JB. The Bidirectional Movement of the Frontalis Muscle: Introducing the Line of Convergence and Its Potential Clinical Relevance. Plast Reconstr Surg. 2020 May;145(5):1155-1162. doi: 10.1097/PRS.0000000000006756. PMID: 32332530. | 42 |
| 47 | Tierney EP, Hanke CW, Watkins L. Treatment of lower eyelid rhytids and laxity with ablative fractionated carbon-dioxide laser resurfacing: Case series and review of the literature. J Am Acad Dermatol. 2011 Apr;64(4):730-40. doi: 10.1016/j.jaad.2010.04.023. PMID: 21414497. | 42 |
| 48 | Joseph AW, Ishii L, Joseph SS, Smith JI, Su P, Bater K, Byrne P, Boahene K, Papel I, Kontis T, Douglas R, Nelson CC, Ishii M. Prevalence of Body Dysmorphic Disorder and Surgeon Diagnostic Accuracy in Facial Plastic and Oculoplastic Surgery Clinics. JAMA Facial Plast Surg. 2017 Jul 1;19(4):269-274. doi: 10.1001/jamafacial.2016.1535. PMID: 27930752; PMCID: PMC5543317. | 41 |
| 49 | Sharma P, Czyz CN, Wulc AE. Investigating the efficacy of vibration anesthesia to reduce pain from cosmetic botulinum toxin injections. Aesthet Surg J. 2011 Nov;31(8):966-71. doi: 10.1177/1090820X11422809. Epub 2011 Oct 14. PMID: 22001341. | 41 |
| 50 | Skaat A, Fabian D, Spierer A, Rosen N, Rosner M, Ben Simon GJ. Congenital ptosis repair-surgical, cosmetic, and functional outcome: a report of 162 cases. Can J Ophthalmol. 2013 Apr;48(2):93-8. doi: 10.1016/j.jcjo.2012.09.010. Erratum in: Can J Ophthalmol. 2013 Jun;48(3):220. Fabian, Ido Didi [corrected to Fabian, Didi]. PMID: 23561601. | 40 |
| 51 | Tripathi A, Haslett R, Marsh IB. Strabismus surgery: adjustable sutures-good for all? Eye (Lond). 2003 Aug;17(6):739-42. doi: 10.1038/sj.eye.6700465. PMID: 12928687. | 40 |
| 52 | Beckingsale PS, Sullivan TJ, Wong VA, Oley C. Blepharophimosis: a recommendation for early surgery in patients with severe ptosis. Clin Exp Ophthalmol. 2003 Apr;31(2):138-42. doi: 10.1046/j.1442-9071.2003.00621.x. PMID: 12648048. | 39 |
| 53 | Foster JA, Barnhorst D, Papay F, Oh PM, Wulc AE. The use of botulinum A toxin to ameliorate facial kinetic frown lines. Ophthalmology. 1996 Apr;103(4):618-22. doi: 10.1016/s0161-6420(96)30644-1. PMID: 8618761. | 39 |
| 54 | Park KY, Hyun MY, Jeong SY, Kim BJ, Kim MN, Hong CK. Botulinum toxin for the treatment of refractory erythema and flushing of rosacea. Dermatology. 2015;230(4):299-301. doi: 10.1159/000368773. Epub 2015 Mar 3. PMID: 25765295. | 38 |
| 55 | Georgescu D, Vagefi MR, McMullan TF, McCann JD, Anderson RL. Upper eyelid myectomy in blepharospasm with associated apraxia of lid opening. Am J Ophthalmol. 2008 Mar;145(3):541-547. doi: 10.1016/j.ajo.2007.10.017. Epub 2008 Jan 11. PMID: 18191096. | 37 |
| 56 | Dutta S, Lorenz HP, Albanese CT. Endoscopic excision of benign forehead masses: a novel approach for pediatric general surgeons. J Pediatr Surg. 2006 Nov;41(11):1874-8. doi: 10.1016/j.jpedsurg.2006.06.047. PMID: 17101362. | 36 |
| 57 | Yuraitis M, Jacob CI. Botulinum toxin for the treatment of facial flushing. Dermatol Surg. 2004 Jan;30(1):102-4. doi: 10.1111/j.1524-4725.2004.30017.x. PMID: 14692937. | 36 |
| 58 | Frankel AS, Kamer FM. The effect of blepharoplasty on eyebrow position. Arch Otolaryngol Head Neck Surg. 1997;123(4):393-396. doi:10.1001/archotol.1997.01900040027004 | 36 |
| 59 | Hamawy AH, Farkas JP, Fagien S, Rohrich RJ. Preventing and managing dry eyes after periorbital surgery: a retrospective review. Plast Reconstr Surg. 2009;123(1):353-359. doi:10.1097/PRS.0b013e31819346ea | 35 |
| 60 | Kim YS, Roh TS, Yoo WM, Tark KC, Kim J. Infrabrow excision blepharoplasty: applications and outcomes in upper blepharoplasty in Asian women. Plast Reconstr Surg. 2008;122(4):1199-1205. doi:10.1097/PRS.0b013e3181858fc0 | 35 |
| 61 | Mohadjer Y, Holds JB. Cosmetic lower eyelid blepharoplasty with fat repositioning via intra-SOOF dissection: surgical technique and initial outcomes. Ophthalmic Plast Reconstr Surg. 2006;22(6):409-413. doi:10.1097/01.iop.0000243607.36661.be | 35 |
| 62 | Lee MC, Chang CS, Huang YL, et al. Treatment of melasma with mixed parameters of 1,064-nm Q-switched Nd:YAG laser toning and an enhanced effect of ultrasonic application of vitamin C: a split-face study. Lasers Med Sci. 2015;30(1):159-163. doi:10.1007/s10103-014-1608-2 | 34 |
| 63 | DeFatta RJ, Krishna S, Williams EF 3rd. Pulsed-dye laser for treating ecchymoses after facial cosmetic procedures. Arch Facial Plast Surg. 2009;11(2):99-103. doi:10.1001/archfacial.2008.538 | 34 |
| 64 | Becker-Wegerich PM, Rauch L, Ruzicka T. Botulinum toxin A: successful décolleté rejuvenation. Dermatol Surg. 2002;28(2):168-171. doi:10.1046/j.1524-4725.2002.01116.x | 34 |
| 65 | Harley RD, Nelson LB, Flanagan JC, Calhoun JH. Ocular motility disturbances following cosmetic blepharoplasty. Arch Ophthalmol. 1986;104(4):542-544. doi:10.1001/archopht.1986.01050160098021 | 33 |
| 66 | Ozkan SB, Can D, Söylev MF, Arsan AK, Duman S. Chemodenervation in treatment of upper eyelid retraction. Ophthalmologica. 1997;211(6):387-90. doi: 10.1159/000310837. PMID: 9380360. | 33 |
| 67 | Cruz AA, Akaishi PM, Mendonça AK, Bernadini F, Devoto M, Garcia DM. Supramaximal levator resection for unilateral congenital ptosis: cosmetic and functional results. Ophthalmic Plast Reconstr Surg. 2014 Sep-Oct;30(5):366-71. doi: 10.1097/IOP.0000000000000105. PMID: 24759292. | 32 |
| 68 | Kapoor R, Shome D, Jain V, Dikshit R. Facial rejuvenation after intradermal botulinum toxin: is it really the botulinum toxin or is it the pricks? Dermatol Surg. 2010 Dec;36 Suppl 4:2098-105. doi: 10.1111/j.1524-4725.2010.01703.x. PMID: 21134041. | 32 |
| 69 | Goldberg RA, Li TG. Postoperative infection with group A beta-hemolytic Streptococcus after blepharoplasty. Am J Ophthalmol. 2002 Dec;134(6):908-10. doi: 10.1016/s0002-9394(02)01848-2. PMID: 12470763. | 32 |
| 70 | Kotlus BS, Heringer DM, Dryden RM. Evaluation of homeopathic Arnica montana for ecchymosis after upper blepharoplasty: a placebo-controlled, randomized, double-blind study. Ophthalmic Plast Reconstr Surg. 2010 Nov-Dec;26(6):395-7. doi: 10.1097/IOP.0b013e3181cd93be. PMID: 20683279. | 31 |
| 71 | Steinsapir KD, Rootman D, Wulc A, Hwang C. Cosmetic Microdroplet Botulinum Toxin A Forehead Lift: A New Treatment Paradigm. Ophthalmic Plast Reconstr Surg. 2015;31(4):263-268. doi:10.1097/IOP.0000000000000282 | 30 |
| 72 | Carruthers A, Bogle M, Carruthers JD, et al. A randomized, evaluator-blinded, two-center study of the safety and effect of volume on the diffusion and efficacy of botulinum toxin type A in the treatment of lateral orbital rhytides. Dermatol Surg. 2007;33(5):567-571. doi:10.1111/j.1524-4725.2007.33115.x | 30 |
| 73 | Taban M, Nakra T, Hwang C, et al. Aesthetic lateral canthoplasty. Ophthalmic Plast Reconstr Surg. 2010;26(3):190-194. doi:10.1097/IOP.0b013e3181baa23f | 29 |
| 74 | Li FC, Ma LH. Double eyelid blepharoplasty incorporating epicanthoplasty using Y-V advancement procedure. J Plast Reconstr Aesthet Surg. 2008;61(8):901-905. doi:10.1016/j.bjps.2007.05.008 | 29 |
| 75 | Carter SR, Stewart JM, Khan J, et al. Infection after blepharoplasty with and without carbon dioxide laser resurfacing. Ophthalmology. 2003;110(7):1430-1432. doi:10.1016/S0161-6420(03)00447-0 | 29 |
| 76 | Lapiere JC, Aasi S, Cook B, Montalvo A. Successful correction of depressed scars of the forehead secondary to trauma and morphea en coup de sabre by en bloc autologous dermal fat graft. Dermatol Surg. 2000;26(8):793-797. doi:10.1046/j.1524-4725.2000.00073.x | 29 |
| 77 | Ellis DA, Tan AK. Cosmetic upper-facial rejuvenation with botulinum. J Otolaryngol. 1997;26(2):92-96. | 29 |
| 78 | Saonanon P, Sithanon S. External Levator Advancement versus Müller Muscle-Conjunctival Resection for Aponeurotic Blepharoptosis: A Randomized Clinical Trial. Plast Reconstr Surg. 2018;141(2):213e-219e. doi:10.1097/PRS.0000000000004063 | 28 |
| 79 | Patel V, Salam A, Malhotra R. Posterior approach white line advancement ptosis repair: the evolving posterior approach to ptosis surgery. Br J Ophthalmol. 2010;94(11):1513-1518. doi:10.1136/bjo.2009.172353 | 28 |
| 80 | Taylor A, Strike PW, Tyers AG. Blepharophimosis-ptosis-epicanthus inversus syndrome: objective analysis of surgical outcome in patients from a single unit. Clin Exp Ophthalmol. 2007;35(3):262-269. doi:10.1111/j.1442-9071.2006.01448.x | 28 |
| 81 | Kim YW, Park HJ, Kim S. Secondary correction of unsatisfactory blepharoplasty: removing multilaminated septal structures and grafting of preaponeurotic fat. Plast Reconstr Surg. 2000;106(6):1399-1406. doi:10.1097/00006534-200011000-00030 | 28 |
| 82 | Huang T. Reduction of lower palpebral bulge by plicating attenuated orbital septa: a technical modification in cosmetic blepharoplasty. Plast Reconstr Surg. 2000;105(7):2552-2560. doi:10.1097/00006534-200006000-00040 | 28 |
| 83 | Kim SH, Lee SJ, Lee JW, Jeong HS, Suh IS. Clinical trial to evaluate the efficacy of botulinum toxin type A injection for reducing scars in patients with forehead laceration: A double-blinded, randomized controlled study. Medicine (Baltimore). 2019;98(34):e16952. doi:10.1097/MD.0000000000016952 | 27 |
| 84 | Ruiz-Esparza J. Noninvasive lower eyelid blepharoplasty: a new technique using nonablative radiofrequency on periorbital skin. Dermatol Surg. 2004;30(2 Pt 1):125-129. doi:10.1111/j.1524-4725.2004.30052.x | 27 |
| 85 | Ghabrial R, Lisman RD, Kane MA, Milite J, Richards R. Diplopia following transconjunctival blepharoplasty. Plast Reconstr Surg. 1998;102(4):1219-1225. doi:10.1097/00006534-199809040-00049 | 27 |
| 86 | Han K, Kang J. Tripartite frontalis muscle flap transposition for blepharoptosis. Ann Plast Surg. 1993;30(3):224-232. doi:10.1097/00000637-199303000-00005 | 27 |
| 87 | Syniuta LA, Goldberg RA, Thacker NM, Rosenbaum AL. Acquired strabismus following cosmetic blepharoplasty. Plast Reconstr Surg. 2003;111(6):2053-2059. doi:10.1097/01.PRS.0000056840.61348.35 | 25 |
| 88 | Schwarze HP, Giordano-Labadie F, Loche F, Gorguet MB, Bazex J. Delayed-hypersensitivity granulomatous reaction induced by blepharopigmentation with aluminum-silicate. J Am Acad Dermatol. 2000;42(5 Pt 2):888-891. doi:10.1016/s0190-9622(00)90264-0 | 25 |
| 89 | Kelly PW, May DR. Central retinal artery occlusion following cosmetic blepharoplasty. Br J Ophthalmol. 1980;64(12):918-922. doi:10.1136/bjo.64.12.918 | 25 |
| 90 | Steinsapir KD, Rootman D, Wulc A, Hwang C. Cosmetic Microdroplet Botulinum Toxin A Forehead Lift: A New Treatment Paradigm. Ophthalmic Plast Reconstr Surg. 2015;31(4):263-268. doi:10.1097/IOP.0000000000000282 | 30 |
| 91 | Prager W, Rappl T. Phase IV study comparing incobotulinumtoxinA and onabotulinumtoxinA using a 1:1.5 dose-conversion ratio for the treatment of glabellar frown lines. J Cosmet Dermatol. 2012;11(4):267-271. doi:10.1111/jocd.12001 | 30 |
| 92 | Carruthers A, Bogle M, Carruthers JD, et al. A randomized, evaluator-blinded, two-center study of the safety and effect of volume on the diffusion and efficacy of botulinum toxin type A in the treatment of lateral orbital rhytides. Dermatol Surg. 2007;33(5):567-571. doi:10.1111/j.1524-4725.2007.33115.x | 30 |
| 93 | Lowe PL, Patnaik R, Lowe NJ. A comparison of two botulinum type a toxin preparations for the treatment of glabellar lines: double-blind, randomized, pilot study. Dermatol Surg. 2005;31(12):1651-1654. doi:10.2310/6350.2005.31303 | 30 |
| 94 | Taban M, Nakra T, Hwang C, et al. Aesthetic lateral canthoplasty. Ophthalmic Plast Reconstr Surg. 2010;26(3):190-194. doi:10.1097/IOP.0b013e3181baa23f | 30 |
| 95 | Li FC, Ma LH. Double eyelid blepharoplasty incorporating epicanthoplasty using Y-V advancement procedure. J Plast Reconstr Aesthet Surg. 2008;61(8):901-905. doi:10.1016/j.bjps.2007.05.008 | 30 |
| 96 | Carruthers A, Bogle M, Carruthers JD, et al. A randomized, evaluator-blinded, two-center study of the safety and effect of volume on the diffusion and efficacy of botulinum toxin type A in the treatment of lateral orbital rhytides. Dermatol Surg. 2007;33(5):567-571. doi:10.1111/j.1524-4725.2007.33115.x | 30 |
| 97 | Kim SH, Lee SJ, Lee JW, Jeong HS, Suh IS. Clinical trial to evaluate the efficacy of botulinum toxin type A injection for reducing scars in patients with forehead laceration: A double-blinded, randomized controlled study. Medicine (Baltimore). 2019;98(34):e16952. doi:10.1097/MD.0000000000016952 | 29 |
| 98 | Taylor A, Strike PW, Tyers AG. Blepharophimosis-ptosis-epicanthus inversus syndrome: objective analysis of surgical outcome in patients from a single unit. Clin Exp Ophthalmol. 2007;35(3):262-269. doi:10.1111/j.1442-9071.2006.01448.x | 29 |
| 99 | Carter SR, Stewart JM, Khan J, et al. Infection after blepharoplasty with and without carbon dioxide laser resurfacing. Ophthalmology. 2003;110(7):1430-1432. doi:10.1016/S0161-6420(03)00447-0 | 29 |
| 100 | Lapiere JC, Aasi S, Cook B, Montalvo A. Successful correction of depressed scars of the forehead secondary to trauma and morphea en coup de sabre by en bloc autologous dermal fat graft. Dermatol Surg. 2000;26(8):793-797. doi:10.1046/j.1524-4725.2000.00073.x | 29 |
